# Biomaterials Mimicking Mechanobiology: A Specific Design for a Specific Biological Application

**DOI:** 10.3390/ijms251910386

**Published:** 2024-09-26

**Authors:** Leonardo Donati, Maria Luisa Valicenti, Samuele Giannoni, Francesco Morena, Sabata Martino

**Affiliations:** 1 Department of Chemistry, Biology and Biotechnology, Biochemical and Biotechnological Sciences, University of Perugia, 06122 Perugia, Italy; 2 Centro di Eccellenza Materiali Innovativi Nanostrutturati per Applicazioni Chimiche Fisiche e Biomediche (CEMIN), University of Perugia, 06123 Perugia, Italy

**Keywords:** polymers, mechanobiology, mechanical response, properties of polymers, tissue engineering, biotechnological application

## Abstract

Mechanosensing and mechanotransduction pathways between the Extracellular Matrix (ECM) and cells form the essential crosstalk that regulates cell homeostasis, tissue development, morphology, maintenance, and function. Understanding these mechanisms involves creating an appropriate cell support that elicits signals to guide cellular functions. In this context, polymers can serve as ideal molecules for producing biomaterials designed to mimic the characteristics of the ECM, thereby triggering responsive mechanisms that closely resemble those induced by a natural physiological system. The generated specific stimuli depend on the different natural or synthetic origins of the polymers, the chemical composition, the assembly structure, and the physical and surface properties of biomaterials. This review discusses the most widely used polymers and their customization to develop biomaterials with tailored properties. It examines how the characteristics of biomaterials-based polymers can be harnessed to replicate the functions of biological cells, making them suitable for biomedical and biotechnological applications.

## 1. Biochemical Mechanobiology: The Proof of Concept for Biomaterial-Based Polymer–Cell Interaction

**Figure 1:**
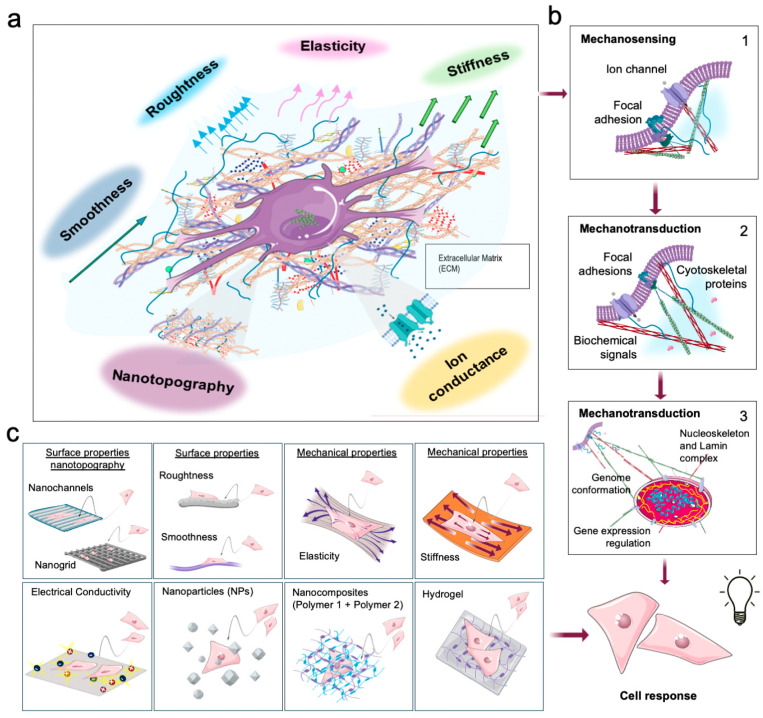
The cartoon illustrates how biomaterials can mimic the characteristics of the Extracellular Matrix (ECM) and the stimuli generated, triggering mechanobiological processes. (**a**) summarizes the different chemical–physical stimuli exerted by ECM at the cellular level. (**b**) schematizes the cellular mechanosensing/mechanotransduction response following ECM stimulation. (**b1**) summarizes the mechanosensing process in which mechanosensory ion channels and Focal Adhesion proteins are involved in perceiving chemical–physical cues. (**b2**) summarizes the process in which the mechanosensing signals are converted into mechanotransduction and biochemical mechanisms by transmembrane, cytoskeletal and soluble proteins, and (**b3**) summarizes the nucleoskeleton (link complex and lamina systems), and the chromatin structure, causing the regulation of gene expression and the cellular response. (**c**) shows the different biomaterial features that elicit similar stimuli to natural ECM, leading to comparable equivalent responsiveness at the cell level.

The field of mechanobiology aims to understand how living cells respond to external biophysical stimuli exerted by the extracellular matrix (ECM) or surrounding fluids [1,2,3,4] (Figure 1a). ECM is a structural macromolecular scaffold that provides biophysical support and drives biochemical signaling, essential for cell homeostasis, tissue development, morphology, maintenance, and function throughout life [1,2,3,4,5].

These events are a consequence of the ECM composition, which consists of (i) solid components, such as proteins (e.g., collagen, elastin, fibronectin), glycosaminoglycans and proteoglycans and (ii) soluble elements such as growth factors and cytokines, mediating the interaction between the ECM and cells. The ECM’s elements confer its geometric conformation, providing topographical stimuli, chemical signals, viscous cues, and mechanical properties (Figure 1a) [6]. The ECM’s mechanical component is mainly due to the elastic fibers, fibrillar collagens, glycosaminoglycans, and associated proteoglycans [7,8]. In this regard, ECM can function as either a “soft material”, deformable under low stress, or a “hard material”, requiring more significant stress for deformation, depending on its composition [7,8].

Biophysical and chemical stimuli elicit dynamic interaction, remodeling the cytoskeleton, triggering a biochemical signal cascade reaching the nucleus, and activating a tailored gene expression program to regulate cell functions and decision-making [2,4,5,9,10,11]. The proteins that perceive and transduce the stimuli mentioned above are known as mechanosensors and include transmembrane proteins (e.g., integrins and ion channels) and intracellular proteins (e.g., Focal Adhesion proteins, cytoskeleton, nucleoskeleton, and specific soluble proteins responsive to physical signals) [2,4,5], organized into molecular complexes that are players, respectively, of mechanosensing and mechanotransduction pathways [2,4,5] (Figure 1b).

When chemical–biophysical cues act on cell membranes, mechanosensing proteins sense the stimuli and relay them to mechanotransduction pathway proteins (Figure 1b) [12,13,14,15,16,17,18]. For example, mechanosensor proteins localized in the cell membrane (e.g., integrins) perceive the stimuli from the ECM microenvironment [12,19] and transmit them to Focal Adhesions, a dynamic complex of various proteins, including Vinculin, Paxillin, and Talin [13]. The complex exhibits rigidity-dependent assembly and turnover, highlighting their mechanosensing/mechanotransduction function in cells. Similarly, the mechanosensing ion channels Piezo 1 and Piezo 2 establish an interplay between the integrin-focal adhesion-actin axis and calcium signaling and convert the inputs into cellular responses (Figure 1b) [16,17,18]. At this stage, the above signal is converted into biochemical pathways transduced by the mechanotransducer proteins and transmitted to the cell cytoskeleton components and related proteins (Figure 1b) [2,4,5]. For instance, Filamin proteins, members of actin-linking proteins, act as a direct organizer of F-actin filaments, interacting with signaling proteins in a force-dependent manner. Moreover, primary cilia microtubule-based structures, Polycystin-1 and α-catenin protein, play crucial roles in the mechanosensing and mechanotransduction of cells to their mechanical environment via the TAZ pathway [20]. The effect of ECM cues is translated by the cell’s cytosolic environment to the nucleus, where they modulate chromatin conformation and gene expression (Figure 1b) [2,21,22,23,24]. In that process, the mechanosensor proteins YAP/TAZ, play a role in transmitting external mechanical signals to the nucleus, influencing cell behavior based on environmental cues like stiffness and topographical organization [23,25].

The importance of mechanosensing and mechanotransduction pathways in cell function is underscored by increasing evidence linking alterations in these biochemical routes to disease development and progression [23], including neurodegenerative diseases [23,26] such as Alzheimer’s and Parkinson’s [27,28], cancer, fibrosis, cardiovascular diseases, and musculoskeletal disorders [26,29]. Understanding the biochemical mechanobiology mechanisms between the ECM and cells enables the development of molecular tools to modulate cellular responses to biophysical cues in health and disease. In this context, polymers can serve as the ideal tool for creating biomaterials designed to mimic the characteristics of the ECM, thereby triggering response mechanisms that closely resemble those induced by a natural physiological system (Figure 1c) [2,21,24].

In the following paragraphs, we review the most widely used polymers, how they are tailored to generate biomaterials with designed properties, and discuss how the properties of biomaterial-based polymers can be applied to mimic the ECM properties with the consequent biological functions.

## 2. Biomaterial-Based Polymers: Overview

Natural or synthetic polymers with biocompatibility characteristics are ideal for generating biomaterials for biotechnological and nanobiotechnological applications in health and industry [30,31,32,33,34,35,36,37,38,39,40] (Table 1 and Table 2).

Table 1 summarizes the major types of natural polymers and their biomedical applications. Natural polymers are ideal for this, because they interact with tissues and cells without being treated as foreign bodies. They are used to build biomaterial with films or scaffold structures that enhance cell growth and tissue formation and to generate envelopes for encapsulation in therapeutic and diagnostic applications [41,42].

Synthetic polymers are used extensively, due to their structural and mechanical properties, reproducibility, cost-effectiveness, and customizable compositions (e.g., high flexibility in chemical modifications and molecular change) [43,44]. Another advantage is the modulation of biodegradability [45], which is suitable for both tissue regeneration or implants for dental reconstruction, sutures, and contact lenses [46,47] (Table 2).

Synthetic polymers might have limitations, such as reduced cell attachment compared to natural polymers, potential immune responses, and toxicity [48].

**Table 1:** List of the main natural polymers, with the indication of the repeat unit, the source of origin, possible biomaterials’ structure, and their applications.

Polymer	Repeat Unit	Source	Biomaterial Structures	Applications	Reference
Alginate	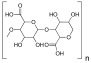 Mannuronic Acid + 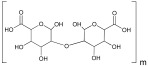 Glucuronic Acid	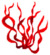 Seaweed	Hydrogels, Scaffolds	Hydrogel for bone tissue engineering application;	[49]
				Hydrogel for hair follicle regeneration;	[50]
				Hydrogel for intervertebral disc regeneration;	[51]
				Scaffold for the treatment of local breast cancer;	[52]
				Scaffold for mesenchymal stem cell cardiac therapy;	[53]
				Films for active packaging applications.	[54]
Cellulose	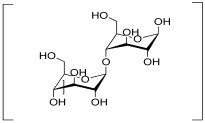 Cellobiose	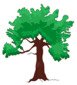 Plants	Nanofibers, Hydrogels, Nanoparticles, Scaffolds	Scaffold for bone regeneration;	[55,56]
				Scaffold for localized drug delivery;	[57]
				Nanoparticles as antibacterial agents;	[58]
				Nanofibers for skin tissue engineering;	[59]
				Nanofibers’ drug delivery;	[59]
				Hydrogel for wound dressing;	[60]
				Hydrogel for bleeding control;	[60]
				Hydrogel for cartilage and neural tissue engineering.	[61]
Chitin	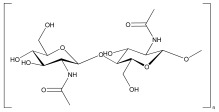 N-Acetylglucosamine	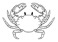 Arthropods, mushrooms and algae	Hydrogels, Scaffolds, Nanomaterials	Hydrogel for cartilage regeneration;	[62]
				Hydrogel and nanoparticles for drug delivery;	[63]
				Nanofibers and hydrogel for wound healing;	[64,65]
				Scaffold for neural tissue;	[66]
				Nanoparticles for cancer treatment.	[67]
Collagen	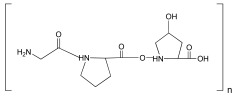 Glycine-Proline-Hydroxyproline	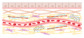 Extracellular matrix (ECM)	Hydrogels, Scaffolds	Scaffold for bone repair;	[68]
				Scaffold for Achilles tendinopathy;	[69]
				Scaffolds for laryngeal cartilage repair;	[70]
				Hydrogel for accelerated diabetic wound-healing;	[71]
				Hydrogel for aging skin rejuvenation.	[72]
Hyaluronic acid	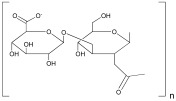 Glucuronic Acid–*N*-Acetylglucosamine	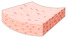 Animal tissues	Hydrogels, Scaffolds, Nanoparticles	Nanoparticles for atherosclerosis;	[73]
				Nanoparticles for drug delivery;	[74]
				Hydrogel and nanoparticles for osteoarthritis;	[75,76]
				Hydrogel for cartilage repair;	[77]
				Combination of hyaluronic acid solution and contact lenses for ophthalmology application.	[78]
Lignin	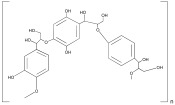 Phenylpropanoid unit	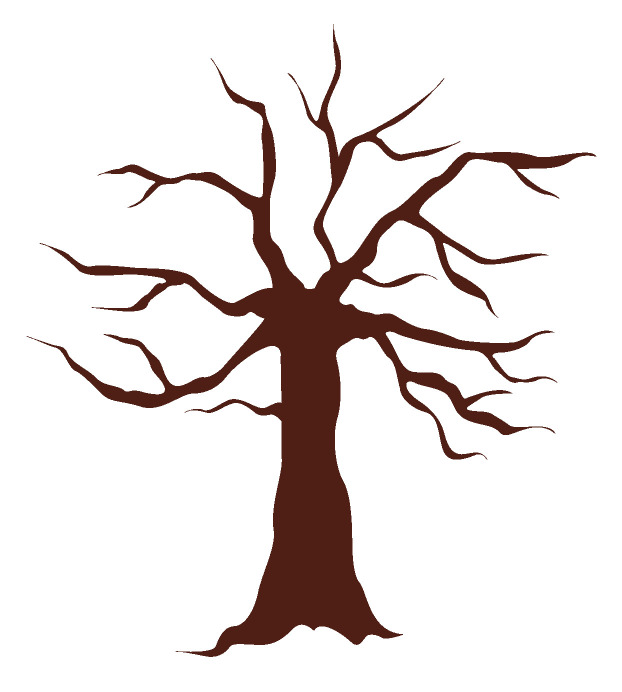 Plant	Hydrogels, Nanoparticles,	Hydrogel for wound healing;	[79]
				Hydrogel for cell immobilization;	[80]
				Nanoparticles for oral drug delivery;	[81]
				Nanoparticles for bone repair;	[82]
				Nanoparticles for cartilage repair;	[82]
				Microparticles for bioplastic generation.	[83]
Silk	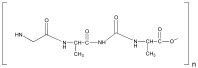 Glycine-Alanine-Glycine-Alanine	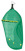 Silkworm cocoons, spiders	Scaffold, Film, Nanoparticles	Scaffolds for bone tissue engineering;	[84]
				Scaffolds for meniscus tissue engineering;	[85]
				Scaffolds for thymus bioengineering;	[86]
				Nanoparticles for drug delivery;	[87]
				Films for wearable biosensors.	[88]

The combination of natural and synthetic polymers, as well as the generation of synthetic-modified polymers (Table 2) makes it possible to create new biomaterials that possess both the complex functionalities of natural polymers and the scalability of synthetic polymers, including modification of their mechanical and physical properties, improving their overall performance [89,90,91,92,93] and the limitation of the original polymers.

Both natural and synthetic polymers can be improved through material libraries, generating modular and supramolecular interactions, which are necessary for the creation of supramolecular aggregates with the ability to mimic ECM [94].

For example, natural and synthetic hydrogels can be used for the generation of supramolecular interactions with proteins, peptides, and other polymers giving them characteristics that mimic ECM (e.g., hybrid hydrogel BSA-polyelectrolytes; alginate/PEG) [94].

It has also been shown that a natural coating of the polymeric Bisurea (BU) material with basement membrane proteins, laminin, and collagen IV, combined with catechol, induces the formation of renal epithelial monolayers [95].

**Table 2:** List of the main synthetic polymers with the indication of the repeat unit, the source of origin, possible biomaterial structures, and their applications.

Polymer	Repeat Unit	Source	Biomaterial Structures	Applications	Reference
Graphene oxide	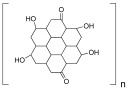 Graphite Oxide	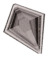 Graphite	Scaffolds, Nanoparticles, Hydrogels, 3D-Bioprinting	Scaffolds for bone tissue engineering;	[96]
				Scaffolds for cardiac tissue engineering;	[97]
				Scaffold for controlled differentiation of human neural progenitor cells;	[98]
				Nanocomposites for endodontic treatments;	[99]
				Hydrogels for microfluidic 3D printing.	[100]
Polyacrylic acid (PAA)	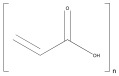 Acrylic Acid	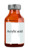 Acrylic acid	Hydrogels, Scaffolds	Hydrogel for anticancer drug release;	[101]
				Hydrogel as an adhesive for medical technology;	[102]
				Scaffold for bone regeneration.	[103]
Polycaprolactone (PCL)	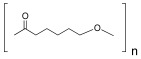 Caprolactone	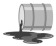 Crude oil	Scaffolds, Nanoparticles, Hydrogels	Scaffolds for bone cancer applications;	[104]
				Hydrogels for tendon tissue engineering;	[105]
				Hydrogels for promoting osteogenic differentiation of adipose-derived stem cells;	[106]
				Scaffold for osteogenic differentiation;	[107]
				Implants for cranial reconstruction after burr hole trephination.	[108]
Polyethylene glycol (PEG)	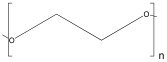 Ethylene Glycol	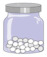 Ethylene	Scaffolds, Hydrogels	Hydrogels for cell proliferation and spreading;	[109]
				Hydrogels support human PSC pluripotency and morphogenesis;	[110]
				Hydrogel for wound care management;	[111]
				Scaffolds with boosted in vitro osteogenic ability;	[112]
				Scaffold-based drug delivery in oral cancer treatment.	[113]
Polylactide (PLA)	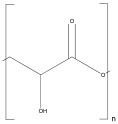 Lactic Acid	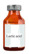 Lactic acid	Scaffolds, Nanocomposites, Biofilms, Hydrogel	Scaffold for bone tissue engineering;	[114]
				Biofilms for improved in vitro bioactivity and stem cell adhesion;	[115]
				Hydrogel promotes diabetic wound healing;	[116]
				Scaffolds promote cell alignment and differentiation;	[117]
				Scaffold for the biological properties of human dental pulp stem cells.	[118]
Polylactide-co-glycol (PLGA)	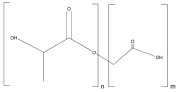 Lactic Acid–Glycolic Acid	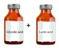 Glycolic acid + Lactic acid	Scaffolds, Hydrogels, Nanoparticles	Nanoparticles for drug delivery;	[119]
				Scaffolds for bone regeneration;	[120]
				Scaffolds for corneal regeneration;	[121]
				Hydrogels as a treatment for osteomyelitis;	[122]
				Scaffolds for cardiac tissue engineering.	[123]
				Membrane for generation of biodegradable stent.	[124]
Polyhydroxybutyrate (PHB)	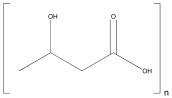 3-Hydroxybutyric acid	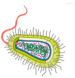 Prokaryotes	Scaffolds, Nanocomposites, Hydrogels	Scaffolds for bone tissue engineering;	[125]
				Scaffolds for peripheral nerve regeneration;	[126]
				Nanocomposites for bone tissue engineering;	[127]
				Fibers for textile applications.	[128]
Polyglycolic acid (PGA)	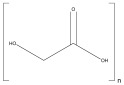 Glycolic Acid	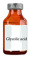 Glycolic acid	Scaffold, Hydrogel	Scaffold for bone tissue engineering;	[129]
				Scaffold for irreparable meniscal tear;	[130]
				Scaffolds to mimic human ear cartilage;	[131]
				Hydrogels in cardiac regeneration.	[132]

### 2.1. Biomaterial-Based Polymer Structure Design

#### 2.1.1. Films

The film structure offers advantages for generating biomaterials with tunable properties such as morphology toughness [133], large-scale processability, and optical, mechanical, electromagnetic, and thermal properties [134], which make them highly sought-after for industry, including agricultural [33,135], food [136], pharmaceutics [137,138], bioengineering, robotics, and bioelectronics [139] (Table 1 and Table 2).

The addition of nanofiller, the monomers units used, the chemical composition, and the film thickness, are parameters that influence the synthesis process and biomaterial properties [140]. Moreover, combining different types of polymers generates micro/nanocomposites with improved characteristics and properties [141,142,143,144]. For instance, the controlled open-loop polymerization technique forming composite films allows the length of the polymer blocks to be adjusted to influence the properties of the resulting films. The functionalization of surfaces with polymer brushes through surface atom transfer radical polymerization offers potential applications as anti-fouling coatings in biological environments [141,142].

#### 2.1.2. Scaffolds

The scaffold structure provides biomaterials that can replicate the properties of native tissues, providing a three-dimensional structure that supports cell proliferation and differentiation and tissue regeneration, resembling the extracellular matrix of various tissues [141,142,143,144] (Table 1 and Table 2). For instance, the use of natural polymers such as gelatin and chitosan in scaffolds mimics the extracellular matrix, promoting cell growth [56,145,146,147], while, among synthetic polymers, polycaprolactone is a popular choice for scaffold fabrication due to its mechanical properties, biodegradability, and solubility [107].

The characteristics and functionality of scaffolds are significantly influenced by the fabrication techniques used [125,148,149]. The fabrication of scaffolds using the Two-photon Polymerization technique has enabled the creation of highly detailed structures at the micro and nanoscale [150]. Melt Electrowinning enables the precise deposition of biocompatible polymers in a layered manner for application [151]. Moreover, hybrid-forming techniques combining traditional methods and newer technologies [152] or nanoparticle integration [153] can help to tailor polymeric-scaffold structure characteristics and functionality. Templating with high internal-phase emulsions also produces porous polymer scaffolds with interconnected porosity [154]. Combining 3D printing with Gas Foaming techniques enables the obtaining of scaffolds tailored in dimensions, geometry, and mechanical strength conducive to cell growth.

Three-dimensional bioprinting addressed the limitations of traditional 2D platforms by enabling the fabrication of scaffolds that mimic the natural environment of tissues and organs [155,156,157,158]. The evolution of 3D bioprinting has also been influenced by technological advances such as machine learning, improving the accuracy and efficiency of printing accurate layered 3D structures [159]. Due to the precise control of biomaterial deposition and the incorporation of biological additives such as cells and biomolecules, bioprinting can fabricate preclinical implants, tissue constructs, and in vitro models tailored to specific needs [160,161,162]. The choice of bio-ink is critical in 3D bioprinting, influencing both the success of printing and the functionality of printed constructs. Silk fibroin-based bio-inks offer standardized protocols for printing soft compositions, addressing stability challenges in long-term culture [163]. Gelatin methacryloyl (GelMA) bio-inks are valued for their thermo-responsive and photo-crosslinking properties, and they are widely used in bioprinting applications [71]. Composite bio-inks like alginate and chitosan are essential for creating organ-on-a-chip models of articular cartilage [164]. Bio-inks derived from the decellularized extracellular matrix (dECM) are studied for their ability to enhance cell growth and promote tissue regeneration [165].

#### 2.1.3. Hydrogel

Hydrogels are polymeric networks with a high affinity for water, formed by the union of smaller or larger monomer units to form a cross-linked structure that is excellent for the growth, development, and study of both monolayer and three-dimensional cell systems [166]. Their high water content, porosity, and intrinsic mechanical tuning make hydrogels particularly attractive as mimics of the ECM [167]. Hydrogels are conventionally defined according to the nature of the polymers composing their chains, the mechanism and subsequent organization of the network assembly, and the length scale of the assembled network [168]. There are natural, synthetic, and hybrid hydrogels; the first class is derived from natural sources and has the intrinsic advantage of being low-cost, non-toxic, and degradable [168], with the limit of poor reproducibility. Synthetic hydrogels, obtained by chemical synthesis and chemical polymerization of networks from artificial compounds, increase the reproducibility of the system but also allow for the obtaining of polymers with well-defined chemical, physical, and mechanical characteristics. Finally, hybrid hydrogels, resulting from the copolymerization of both synthetic and natural monomers, are produced to obtain new biomaterials having improved properties of both constituents (e.g., the combination of hydrogels and porous polymer microparticles is promising for advanced functionality in biomaterial design) [169,170].

#### 2.1.4. Nanoparticles

Designing nanoparticles (NPs) is one of the significant tools in the science of nanomaterials explored in biology and medicine, due to their nanometric size (range from 1 to 100 nm) [171,172]. NPs can be produced by controlled synthesis processes to obtain specific shapes and sizes, as well as imparting various physicochemical properties, including surface charge, the ability to form agglomerates, and the possibility of being functionalized with other bioactive molecules [169,170,173,174,175], which is particularly useful in regenerative medicine [20,173,176,177].

NPs are synthesized through two primary technical methods: the top–down approach involves breaking down larger structures into nanoparticles, and the bottom–up approach builds nanoparticles from smaller components [178,179].

The physical properties of nanoparticles, including crystal structure, size, and shape, can influence their optical properties, affecting their performance in bioimaging applications [180] and functionality. Thus, NPs with selected sizes and structures have been shown to possess robust photoacoustic and photothermal capabilities, making them suitable for applications such as photo theranostics [181].

Noble metal nanoparticles have attracted significant attention, due to their high stability, corrosion resistance, and catalytic activity [182]. These size- and shape-dependent physical and chemical properties of noble metal nanostructures have led to widespread applications in photonics, catalysis, and other fields [183,184]. Furthermore, noble metal nanoparticles with materials such as metal–organic frameworks (MOFs) have created new application opportunities in sonodynamic and photodynamic therapy [185].

## 3. Properties of Biomaterial-Based Polymers

As mentioned, biomaterial-based polymers, due to their unique properties [186], can dictate biomaterial applications in health and biotechnological industries [39,187,188,189,190].

In this section, we discuss the different properties of biomaterial-based polymers, how these are measured, and how cells collect and respond to them (Table 3).

### 3.1. Chemical Properties

The polymers’ chemistry defines the biomaterials’ identity [191,192,193]. Generally, the chemical composition of biomaterials simulates the chemical characteristics of the ECM, providing chemical stimuli comparable to the physiological one, promoting integrin-mediated adhesion and the differentiation of stem cells (Table 3). Thus, the type of polymer, functional groups, and the method used for the synthesis are critical steps for designing biomaterials. For instance, functional groups, such as hydroxyl, carboxyl, amino, ester, phosphate, and sulfonate, enhance the reactivity and biocompatibility of biomaterials, increasing the cell’s adhesion [194]. Similarly, the methods used for generating homopolymers or heteropolymers could leverage the final chemical properties [195]. Furthermore, the intramolecular forces, such as hydrogen bonds, covalent bonds, and Van der Waals interactions, generated among the functional groups, influence the chemical structure of the biomaterials and their capability of providing chemical stimuli and interacting with cells [196].

The characterization of the chemical composition involves several techniques, such as Fourier-Transform Infrared Spectroscopy, X-ray Diffraction, and Raman Microspectroscopy, used for identifying the molecular composition, crystal structure, and degree of crystallinity of polymers [197,198,199].

### 3.2. Physical Properties

Mechanical, electrical, and thermal properties can be distinguished at the level of physical properties (Table 3).

The mechanical characteristics of biomaterials encompass a range of properties, including tensile strength, Young’s modulus, viscoelasticity, and stiffness [200]. These properties are influenced by the polymer composition, methods used in processing, and the presence of fillers or reinforcements [201,202]. Mechanical stimuli provided to cells can significantly change their morphological structure, leading to various biological responses, such as stem cell differentiation. This process is mediated by several mechanotransducer proteins, including YAP and TAZ proteins, Focal Adhesion Kinase (which promotes adhesion and interaction with actomyosin, facilitating the cytoskeletal network reorganization), and GTPases activity (which regulates cell migration and activation of ionic channels, such as Piezo 1) (Table 3).

Rheological analyses give information about the mechanical properties of biomaterials, providing valuable insights into their viscoelastic properties [203,204,205], such as the crosslink density in polymer-based hydrogels, which significantly influences their mechanical properties [206]. Non-destructive and contact-free methods, such as Dynamic Light Scattering and Brillouin Spectroscopy, can also analyze the mechanical properties [207,208,209,210,211,212,213]. Additionally, the mathematical models Voigt and Burger’s and the fractionate derivative model are useful in predicting the deformation of the biomaterial under different conditions [207,208,209,210,211,212,213].

The electrical properties of biomaterials include conductivity, ion conductance, and piezoelectricity [214]. The ability of biomaterials to conduct electricity activates several signaling pathways at the cellular level, including MAPK/ERK, PI3K/Akt, NF-kB, Wnt/β-catenin, and Notch. This activation promotes the proliferation and differentiation of cardiac and neural cells and stimulates voltage-dependent ion channels, which enhance electrophysiological activity (Table 3). Alternate Current Impedance Spectroscopy [215] and Dielectric Relaxational Spectroscopy are the main techniques used to measure the complex dielectric permittivity of polymers [216]. Another technique is Kelvin Probe Force Microscopy, which enables the precise mapping of surface potential by measuring the contact potential difference between the tip of an atomic force microscope and the sample surface [217].

The thermal properties of biomaterials and their composites refer to their behavior under different temperature conditions [218,219,220]. The principal parameter involved is thermal conductivity, which is the ability of biomaterials to conduct heat. Biomaterials’ ability to minimize thermal fluctuation is necessary for biological applications, as it reduces cell thermal stress, decreases ROS production, and enhances mitochondrial function (Table 3). The thermal properties are measured by Differential Scanning Calorimetry, Thermogravimetric analysis, and Laser Flash analysis [221,222,223,224,225,226,227].

Computational modeling can be utilized to define and predict the properties of polymers before synthesis, to achieve specific designs and stimuli. Molecular Dynamics (MD) simulations are essential to progress the biomaterial design and mimic the sophisticated features of the ECM. These tools can be used to reproduce the molecular structure and mechanical properties of synthetic polymer networks for both softness, hardness, and for mechanical performance [228]. These tunable properties are essential for the development of biomaterials that match the unique viscoelastic nature of native tissues, making them affordable in regenerative medicine and tissue engineering (TE) [228]. Computational methods can also be used as a tool to understand how material properties affect cell behavior and to predict biocompatibility and function [229]. With MD simulations, it is possible to evaluate how changes in polymer chains or crosslinking density impact the material capacity to enable cell attachment, proliferation, and differentiation. These computational methods not only significantly improve the accuracy of biomaterial design, but also expedite the process through which new materials are designed without the requirement to subject them to countless experimental trials [230].

### 3.3. Surface Properties of Polymer Films and Scaffolds

Surface properties of biomaterials include wettability, roughness/smoothness, porosity, and micro- and nano-topography [231].

X-ray Diffraction, Fourier-Transform IR Spectroscopy, Scanning Electron Microscopy, Atom Force Microscopy, and Micro-Computed Tomography are the most frequent instruments used for the analyses of roughness and porosity of biomaterials [232,233,234,235]. The wettability of a polymer is usually evaluated through Water Contact Angle measurements [236] or in silico analysis with Molecular Dynamic simulations, which provide insights into properties like water absorption on polymer surfaces and interactions with solid surfaces [237]. All these analyses also provide information on the degradation time and alterations in the morphology of polymer blends.

The surface properties of polymers generate various deformations in cytoskeletal organization, leading to increased adsorption of extracellular matrix proteins and facilitating integrin-mediated cell adhesion (Table 3). Additionally, different surface characteristics promote specific types of cell differentiation: hydrophilic surfaces enhance osteogenic differentiation, rough surfaces stimulate osteoblast differentiation, porous scaffolds encourage chondrogenic differentiation, and nano-patterned surfaces favor neurite outgrowth (Table 3).

**Table 3:** List of properties of biomaterial involvement in mechanobiology, and the molecular effect on the cells and the biological applications.

Roles in Mechanobiology					
	Properties	Cues	Cell Molecular Response	Biological Applications	Reference
Chemical Properties	Composition	Functional groups, synthesis methods and intramolecular forces determine the ability of biomaterials to simulate the cues derived from the Extracellular Matrix	Increase in the integrin-mediated adhesion	Chemical structure and the inclusion of active biomolecules activate a specific molecular pathway	[238]
			Directing stem cell differentiation and proliferation		[239]
Physical Properties	Tensile Strength, Young’s Modulus, Viscoelasticity, Stiffness	Tensile strength stimulates cells to assume a flattened morphology and generate strong adhesion An elevated Young’s Modulus value stimulates cells to assume a more rounded morphology, with less-pronounced stress fiber Viscoelastic Biomaterials exhibit a different time-depending strain based on the external cues, which affect cell shape, causing an initial spread of cells, but, over time, the cell might relax and adopt more rounded morphology Stiffness, which refers to the resistance to deformation, provides mechanical cues, depending on the proper resistance of biomaterials, which leads to changes in the cell’s shape, adhesion strength, and differentiation fate	Promote activation of Focal Adhesion Kinase (AFK) by facilitating autophosphorylation at Tyrosine 397, generating strong adhesion		[240,241,242]
			Stiff polymers cause the translocation of YAP and TAZ in the nucleus, promoting Osteogenesis	Bone tissue regeneration	[243,244,245,246]
			Stiff polymers cause the translocation of YAP and TAZ in the nucleus, promoting Myogenesis	Skeletal muscle regeneration	[243,244,245,246]
			Soft polymers lead the remaining YAP and TAZ in the cytoplasm, promoting adipogenesis	Generation of adipose tissue for facial and breast reconstructive surgery	[247,248,249,250,251]
			Soft polymers lead the remaining YAP and TAZ in the cytoplasm, promoting neurogenesis	Neural tissue regeneration	[247,248,249,250,251]
			Generation of higher contracting through actomyosin interactions, causing well-defined cytoskeletal network and the tendency of nuclei to be elongated and flattened, promoting Epithelial differentiation	Regeneration of epithelial tissue for airway epithelium development and kidney regeneration	[252,253,254,255,256]
			Stiff substrates promote activation of RhoA GTPase, through its effector Rho-associated kinase (ROCK), facilitating the formation of actin stress fiber modulating the Epithelial-to-Mesenchymal Transition	The Activation of RhoA GTPase and Rac1 GTPase is involved in different proliferation and differentiation pathways; the specificity depends on other characteristics of biomaterials	[257,258]
			Soft substrate favorites the activation of Rac1 GTPase, which promotes the formation of lamellipodia and membrane ruffles, associated with Epithelial-to-Mesenchymal Transition		[257]
			Stiffer substrates cause modification of the cytoskeletal arrangement, causing activation of Piezo channels, allowing the influx of Calcium and Sodium cations that promote osteogenic differentiation	Bone tissue regeneration	[18,246]
			Soft Biomaterials can simulate the action of the Tympanic Membrane, transmitting the vibration to hair cells of the cochlea, activating Mechanical Gated Channels providing the conversion of mechanical stimulus to an electric one	Biodevices for the restoration of tympanic membrane	[259,260]
	Conductivity, Ion Conductance, Piezoelectricity	Conductivity and Piezoelectricity provide electrical cues that simulate the physiological one, promoting differentiation and electrophysiological activity Ion Conductance provides movement of ions, generating ionic cues that stimulate the cell’s proliferation and activity	Activation of MAPK/ERK, PI3K/Akt, and NF-kB, promoting the proliferation of Neuronal cells	Neural tissue regeneration	[261,262,263]
			Activation of MAPK/ERK, PI3K/Akt, and NF-kB, promoting the proliferation of Cardiac cells	Cardiac muscle regeneration	[261,262]
			Activation of MAPK/ERK, Wnt/β-catenin, and Notch signaling, causing the differentiation of Neuronal progenitor stem cells	Neural tissue regeneration	[261]
			Activation of MAPK/ERK, Wnt/β-catenin, and Notch signaling, causing the differentiation of Cardiac progenitor stem cells	Cardiac muscle regeneration	[261,262]
			Activation of TGF-B, BMP, Wnt/β-catenin, and Notch signaling, causing the differentiation of Osteogenic cells	Bone tissue regeneration	[246,262]
			Activation of voltage-gated channels with the enhancement of Synaptic Transmission and Action Potential propagation	Neural function regeneration	[264,265]
	Thermal Conductivity	Thermal conductivity provides the maintenance of uniform temperature, reducing cell stress	Minor stress causes lower levels of ROS and reduced activation of the Heat Shock Response pathway	The thermal conductivity of biomaterials, in combination with other characteristics, allows possible biological application	[266,267,268]
			Regulation of the temperature causes increased mitochondrial functions such as ATP production and electron chain transport		[269,270]
Surface Properties	Wettability, Roughness, Porosity, Micro- and Nano- Topography	Wettability is a surface parameter that indicates if a biomaterial is hydrophilic or hydrophobic; this affects how cells spread, shape themselves, and differentiate The roughness of the biomaterial’s surface influences the spreading of cells and the formation of cellular protrusion The porosity of a biomaterial could create a microenvironment that mimics the natural tissue structure, affecting the cells’ spread, shape, and differentiation Micro- and Nano- Topography refers to the three-dimensional features and texture of biomaterial’s surface, which affects the cells’ morphology and differentiation by influencing cell spread, alignment, and forming of shapes	Increased adsorption of fibronectin, collagen, and lamin promote integrin-mediated binding and Extracellular Matrix production	Generation of extracellular matrix studies model	[271,272,273,274]
			Hydrophilic surfaces enhance the adsorption of Bone Morphogenetic Proteins, fibronectin, and osteopontin, and influence the deposition of calcium ions and the formation of hydroxyapatite crystals, promoting osteogenic differentiation	Bone tissue regeneration	[275]
			Rough surfaces induce a conformational change in the adsorbed proteins such as fibronectin, collagen, and osteopontin, causing the exposition of binding sites, enhancing focal adhesion formation, and promoting osteoblast differentiation	Bone tissue regeneration	[276]
			A porous scaffold mimics the mechanical properties of native cartilage, and also adsorbs and exhibits chondrogenic growth factors such as TGF-B and IGF-1, promoting chondrogenic differentiation	Chondrogenic tissue regeneration	[277,278,279,280,281]
			Nano-patterned surfaces provide topographical cues that influence the organization and dynamics of actin cytoskeleton and microtubules, causing neurite outgrowth	Neural tissue regeneration	[282,283]

## 4. Biomaterial-Based Polymer for Biological Applications

**Figure 2:**
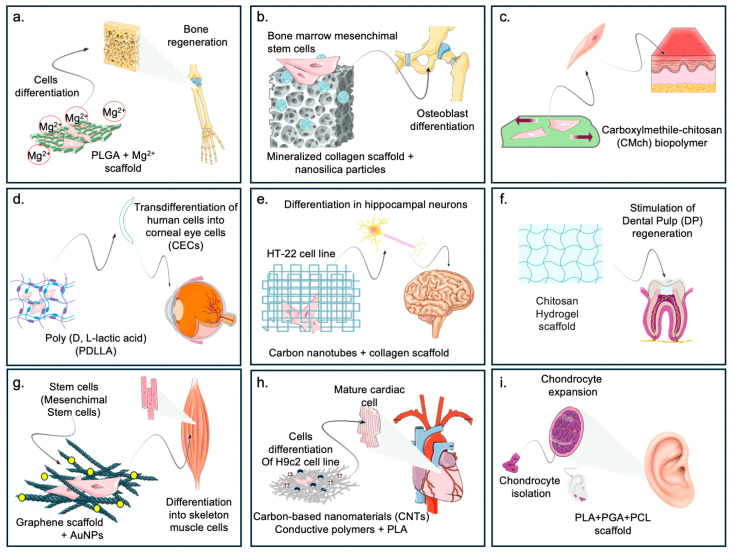
The figure highlights the different fields of application of biomaterials at the biomedical level. (**a**) Cells cultured on magnesium-functionalized biomaterials can be directed toward osteogenic differentiation [285]. (**b**) Cells cultured on composite materials enriched with differentiation factors are directed toward bone tissue regeneration [56]. (**c**) Biomaterials with a specific stiffness can exert forces that drive toward epidermal differentiation. (**d**) PLA enables trans-differentiation of stem cells to corneal cells for corneal reconstitution [286]. (**e**) Stem cells subjected to a given mechanical stimulus can be directed toward neural differentiation by changing their fate [287]. (**f**) Biomaterials implanted at the dental level can stimulate regeneration of the dental pulp after damage [288]. (**g**) shows the use of composite materials (graphene scaffolds + Nanoparticles) that can regenerate skeletal muscle tissue from stem cells [289]. (**h**) The use of a composite material of carbon and PLA enriched with electrical charges enables the differentiation of stem cells to adult myocardial cells, reconstituting possibly damaged heart tissue [139]. (**i**) highlights how the production of a mold made of PLA, PGL, and PCL represents an excellent substrate in which stem cells can grow and differentiate toward cartilage cells, regenerating an auricle [290].

**Figure 3:**
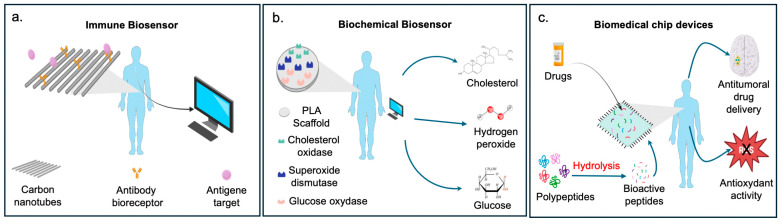
In the figure are some examples of biosensor-based polymers: (**a**) possible use of CTN scaffold as immune biosensor to detect specific target antigen; (**b**) application of PLA scaffold as a biochemical biosensor for the immobilization of specific proteins capable of detecting glucose, hydrogen peroxide, and cholesterol; (**c**) use of biomaterial for drug delivery of antitumoral compounds and as a scaffold to immobilize small bioactive peptides [308,309,310,311,312].

In this section, we discuss the correlation between the specific chemical/physical characteristics of biomaterials, the activated cellular mechanobiological pathways, already introduced in Table 3, and the induced biological responses. These are highlighted in the biomedical application of biomaterials, such as tissue engineering and biodevices (Figure 2 and Figure 3).

### 4.1. Tissue Engineering

Tissue engineering (TE) is a multidisciplinary science dedicated to generating and restoring tissues using the principles of engineering, chemistry, and physics, combined with an understanding and application of the biological sciences and medicine [284]. In TE, biomaterials can serve as a scaffold for treating and repairing different body tissues. Herein, the successful application of biomaterials in the biomedical field is a function of critical characteristics: biocompatibility, biodegradability, specific mechanical properties (e.g., elasticity, stiffness), specific properties at the biological level (e.g., stimulation cell growth, cell migration), and specific structural design, which can stimulate an equally specific response at the cellular, and thus, tissue, level. Examples of biomaterials applied in TE are summarized in Figure 2.

Hydrogel systems are widely studied in the regeneration of new cartilage, due to their unique porous structure, and, most importantly, their similarity to the natural ECM, capable of creating an environment and stimuli as similar as possible to the original tissue that allows cell adhesion, migration, development and differentiation of chondrocytes and osteoblasts, and the passage of nutrients and growth factors [291]. Similarly, the use of collagen I or esterified hyaluronic acid mesh porous scaffolds [292] has been shown to elicit differentiation towards chondrocytes, as evidenced by Extracellular cartilaginous proteoglycan formation, over time. This differentiation can also be achieved through TGF-β and IGF-1 in the porous scaffolds [288] (Table 3).

Biomaterials are equally helpful for the complete reconstruction of an entire cartilaginous organ, as reported in the study by Zhou and co-authors [293], in which the use of 3D PLA-PGA and PCL biomimetic polymers enabled the reconstruction of an entire auricle in patients with Microtia. Specifically, an exact and mirrored replica of the auricle having similar and stable mechanical properties compared to that of healthy tissue was produced through 3D printing techniques. Autologous cartilage cells were placed on the surface of the scaffold and allowed to develop until complete tissue regeneration and the subsequent implantation of the regenerated tissue in the patient [294].

Recent approaches in bone restoration led to the generation of bioactive scaffolds that mimic the natural microenvironment present in natural bone tissue, to provide a substrate like the natural environment. Besides the biological characteristics and mechanical properties, porosity is essential to reaching the bone restoration goal. In silico modeling could help project-specific bone scaffolding supports, guaranteeing the development of new devices for tissue engineering applications. As such, hydroxyapatite shows excellent osteo-inductive properties, thanks to its ability to mimic the natural bone environment, providing an ideal substrate for cell attachment, growth, and development [295]. Biodegradable and biocompatible polymers such as PLA and PCL, alone or in combination, are promising materials generating a specific scaffold explicitly designed for bone restoration in structure and function [296]. Moreover, including osteo-inductive molecules, such as Magnesium (Mg^2+^), might improve the osteogenic potential of PLA-based biomaterials, as demonstrated by the activation of the expression of osteogenic genes [297]. Magnesium-based biodevices for bone repair show a significant advantage over other materials, such as ceramic scaffolds or PLA and PLGA polymers, due to excellent mechanical properties compared to other biodevices, strong osteo-inductive capabilities, and biodegradability [298]. Similar effects were also obtained by using β-Tricalcium phosphate (β-TCP) ceramics or Gelatin methacryloyl (GelMA) polymer [299]. These biomaterials determined a passive mechanical signal that culminates with the translocation of YAP and TAZ within the nucleus [241,243], promoting the osteogenesis process. Modification of the surface characteristics of pure titanium or functionalized titanium (Ti6AL4V) allows the generation of cell protrusions, the formation of new focal adhesion, and osteogenic differentiation, as shown by the expression of bone morphogenetic proteins, fibronectin, and osteopontin and the enhancement of calcium ion deposition and hydroxyapatite crystal formation (Table 3) [287].

Nanocellulose-based (NC) composite materials scaffolds have also proven functional in bone tissue regeneration, generating softer and stiffer tissues, as the scaffold’s mechanical properties can be shaped as needed [300].

The intervention techniques available for the correction of vision defects, to date, make it possible to regain excellent visual abilities by resorting, however, to less invasive surgical practices that require the removal of corneal tissue from a donor and reimplantation in the patient. To date, the technique of in vitro expansion of Corneal Endothelial Cells (CECs) and their subsequent injection, along with scaffold-based Corneal Endothelial Tissue Engineering (CECT) techniques, is the most innovative and cutting-edge technique. In this case, scaffolds are produced from both synthetic and natural polymers, using 3D-printing or electrospinning techniques on which different cell lines, human pluripotent stem or corneal endothelial cells isolated from donors, can be grown, generating a corneal tissue graft that can be transplanted into individuals with defects in this area of the body [56].

Dental pulp regeneration using biomaterials has become increasingly popular, replacing traditionally used intervention techniques such as removing damaged teeth or occluding caries using sealing pastes. There are many biomaterials used today, including collagen, silk fibroin, and chitosan, to reconstitute dental pulp naturally. In dentistry, gelatin-based biomaterials have excellent characteristics, due to their biocompatibility and ability to support the adhesion and growth of this natural polymer’s dental pulp stem cells (DPSCs). Furthermore, they are excellent natural polymers for generating three-dimensional heteropolymer scaffolds with well-defined micro- and macroscopic characteristics like specific tissues and organs [301]. Studies in which heteropolymer scaffolds consisting of gelatin and fibroin were generated have been shown to have chemical–physical and biological characteristics that can promote migration, proliferation, and odontogenic differentiation of DPSCs [302]. The production of biomimetic, chitosan-based scaffolds has successfully pushed dental pulp stem cells toward differentiation into mature cells expressing osteogenic and odontogenic differentiation markers [288].

In vitro TE experiments based on the interaction between stem cells and biomaterials have proved helpful in demonstrating how it can influence a cell’s fate and differentiation, based on mechanical stimulus/cellular response crosstalk. In this sense, human bone marrow progenitor mesenchymal stem cells (hBM-MSCs) were cultured on the cycloaliphatic polyester biomaterial poly (butylene 1,4-cyclohexane dicarboxylate) (PBCE) [287]. The cells respond to mechanical stimuli, rearranging their morphology due to the reorganization of F-actin filaments, assuming a shape like that of neural progenitor cells, confirmed by the expression of elevated levels of neural differentiation markers [287].

Moreover, soft and easily modifiable materials, e.g., polydimethylsiloxane (PDMS), were used to promote neural differentiation of cells from a mechanical perspective [303]. The retention of YAP and TAZ at the cytoplasmic level is controlled by these polymers, which results in differentiation into nerve cells.

In addition, biomaterials that transmit an electrical stimulation, such as Electrospun poly(caprolactone)/gelatin + evaporated AuNps and Silk Fibroin gel-graphene, allow the activation of the MAPK/ERK, PI3K/Akt, and NF-kB pathways, promoting the proliferation of neural cells. Finally, neural differentiation is also dependent on the nano-topographic characteristics of the biomaterial, and, therefore, on the mechanical stimuli that the cell perceives, to which it responds by changing and reorganizing the cytoskeletal actin filaments and microtubules, culminating in the generation of neurite-like growths (Table 3) [247,249,261,282,283].

The concept of mimicking the ECM to influence cellular processes for therapeutic purposes has also been applied in vitro to the reconstitution of skeletal muscle tissue. Stem cells can be used in combination with biomaterials of different natures, allowing them to generate stimuli and thus cellular responses culminating in differentiation into skeletal muscle cells [304]. Graphene and graphene functionalized with chemical oxygen species are two examples of biomaterials that successfully lead to skeletal muscle regeneration due to their strength, tensile strength, and surface characteristics and the ability to stimulate cell adhesion and increase the content of myogenic proteins such as myosin heavy chain and myogenin, thus driving cells to myogenic differentiation [289]. At the molecular level, following a mechanical stimulus, the cell’s mechanosensitive calcium channels are activated, determining a cascade of downstream signals, for the rearrangement of F-actin microfilaments, inhibition of Pax7 expression, and induction of myogenin. During the early stages of cell proliferation, YAP is overexpressed, while during the differentiation phase, YAP activity is inhibited, thanks to the phosphorylation of YAP in serine residue. This event causes its translocation from the nucleus to the cytoplasm and leads to an overexpression of the MyoD protein [305].

Heart tissue disease and damage are increasingly common nowadays, and remain a significant cause of death. The development of electroconductive biomaterials for the reconstitution of a functioning myocardium is one possible avenue for solving the complete reconstitution of this complex tissue. Carbon-based nanomaterials such as carbon nanotubes (CNTs) and graphene have been extensively investigated in cardiac tissue regenerative biomedicine. CNTs exhibit excellent electrical conductivity capabilities and specific surface area, stimulating myocardial differentiation and possible cardiac tissue regeneration [139]. In heart regeneration, Chitosan, a derivative of chitin, has proven helpful in suggesting a potential tool for heart TE regeneration. In addition, Chitosan has proven helpful in the regeneration of bone, dental pulp, and epithelial tissue [306]. Other biomaterials with electrical conductivity, for example, the membrane of Poly-l-Lysine-PANI nanotube membranes, PLCL, and PANI electrospun membranes [307], provide electrical stimuli capable of inducing the restoration of cells functioning from an electrophilic point of view. Molecular-active conductive materials promote the activation of MAPK/ERK, PI3K/Akt, and NF-kB, promoting the regeneration and proliferation of Cardiac cells [261].

In conclusion, the success of TE must recognize the principles governing mechanobiology and, thus mimic the cell/ECM interaction. This is highlighted by the effectiveness of TE application in recent clinical trials which are FDA-approved (Table 4).

### 4.2. Biodevices

Research in biomaterials has also developed innovative tools for health biotechnological applications (Figure 3).

Advances in applications are increasingly moving towards producing wearable electronic devices that can detect and monitor the health of the person wearing them [308,309,310]. This includes so-called biosensors, devices containing biological elements that can specifically bind to a target analyte. These components comprise a bioreceptor and a recognition molecule, which can be an enzyme, protein, antibody, DNA, virus, or similar substances (Figure 3) [313]. Natural and synthetic degradable biomaterials such as starch, gelatin, silk, cellulose, polylactic acid, polyurethane, etc., have been widely explored as building blocks for the construction of disposable or transient electronics [314].

Research in biosensors has led to the development of bioactive CTN polymers specializing in various biosensor functions, including the ability to monitor glucose levels, hydrogen peroxide, cholesterol, and immune-sensing (Figure 3) [313]. In this context, the technology of aptamers [315] represents a further advancement, as they can include single-stranded DNA/RNA oligonucleotides as biosensors.

Neoplastic diseases are one of the leading causes of death worldwide. Traditionally applied therapies have the disadvantage of producing significant side effects in patients. Recently, research has investigated devices that can provide targeted, non-invasive treatment to resolve malignancies (Figure 3). Nanomaterials have gained much attention, due to their specificity and small size. Nanoparticles with a non-spherical shape are potentially very effective in targeted therapy against different types of tumors, being injected into the area of interest and functioning as a drug carrier. This allows surrounding healthy organs and tissues to be left intact. In addition, nanometric biomaterials of natural origin (animal or plant proteins, gelatin, and silk fibroin) offer significant advantages over synthetic ones in the drug delivery system, due to their marked biocompatibility and biodegradability. This makes it possible to avoid further interventions on patients [316]. Moreover, innovative carbon-based nanomaterials have attracted attention, due to their geometrical, electrical, and surface properties, which make them excellent substrates for binding molecules such as antibodies, proteins, or peptides. These make the CNTs a valuable candidate for delivering active biomolecules with enzymatic, antioxidant, and antigen-recognition functions of specific target antigens and nucleic acid molecules.

## 5. Conclusions

Biomaterials, with their unique characteristics of polyhedrality and versatility, represent a thrilling frontier in research. Their properties, intricately linked to the nature of the polymer used (natural, synthetic, and synthetic-modified), the methods of synthesis, and the combination of different polymers, hold huge potential.

By manipulating these processes, with the help of computational predicational tools, we can obtain biomaterials with specific and diverse physical, chemical, and surface properties, opening new avenues in research fields, either in health (tissue engineering, and molecular mechanisms responding to mechano-physical stimuli) or in biotechnological industries (food packaging and antimicrobial devices for the food industry). It should be borne in mind that, in recent years, the polymer sector has gained a foothold in the field of food packaging, thanks to the properties of some of the biomaterials being antimicrobial and sustainable from a biomedical point of view.

The latest research in the field of biomaterials applied to tissue engineering is pushing the boundaries, aiming to produce biodevices that mimic the extracellular environment’s chemical characteristics and mechanical forces. This could revolutionize tissue repair mechanisms, producing mechanical and biochemical events comparable to the native environment. This research argues that a specific design triggers a specific response at the cellular level, and, by shaping the properties of a polymer, we can also alter the generated response. The latest goal to be reached in the field of biomaterials is the surgical/clinical applicability and scalability of these biomaterials, a prospect that is both exciting and promising.

## Figures and Tables

**Table 4:** Examples of FDA approval of clinical trials in TE.

Biomaterial	Clinical Trial	Aim of Study	ID Number
Bioengineered Bilayered Living Cellular Construct	A bioengineered living-cell construct activates an acute wound-healing response in venous leg ulcers	Treatment of Chronic nonhealing venous leg ulcers (VLUs)	NCT01327937 (2017)
Tricalcium Phosphate	EUDRA-CT	Atrophic Nonunion of long bones	NCT02483364 (2020)
Hydroxyapatite + collagen	A multilayer biomaterial for osteochondral regeneration shows superiority vs. microfractures for the treatment of osteochondral lesions in a multicenter randomized trial at 2 years	Assess the benefit provided by a nanostructured collagen–hydroxyapatite (coll-HA) multilayer scaffold for the treatment of chondral and osteochondral knee lesions	NCT01282034 (2021)
Carbon nanomaterials	Carbon nanomaterials for cardiovascular theranostics: promises and challenges	Drug-delivery Biosensor Tissue engineering Immunomodulation	NCT02698163 (2016)
Autologous cartilage cells expanded ex vivo	Autologous chondrocyte implantation (ACI) in the knee: systematic review and economic evaluation	Assess the clinical effectiveness and cost-effectiveness of ACI in chondral defects in the knee, compared with microfracture (MF)	TIG/ACT/01/2000 (2017)
Collagen Alginate Dressing	Omega3 Wound Fish Skin Graft in the Treatment of DFUs	Treatment of diabetic foot ulcers (DFUs)	NCT04133493 (2019 to 2022)
Platelet-Rich Plasma (PRP)	Study on the healing of the partial skin-graft donor site in burn patients	Skin burn regeneration	2016-000968-42 (2016)
Mucopolysaccharides (Hyaluronic acid + Chondroitin sulfate)	Regeneration of ischemic damage in the cardiovascular system using Wharton’s jelly as an unlimited source of mesenchymal stem cells for regenerative medicine	Regeneration of cardiovascular damaged tissue	2016-004684-40 (2018)
Autologous Chondrocyte implantation product	A Clinical Study to Evaluate the Safety and Effectiveness of NOVOCART^®^ 3D Plus Compared to Microfracture in the Treatment of Articular Cartilage Defects of the Knee.	Repair of localized, full-thickness cartilage defects of the femoral condyle (medial, lateral, or trochlea) of 2–6 cm^2^	2011-005798-22 (2012)
